# Narrow Band Filter at 1550 nm Based on Quasi-One-Dimensional Photonic Crystal with a Mirror-Symmetric Heterostructure

**DOI:** 10.3390/ma11071099

**Published:** 2018-06-27

**Authors:** Fang Wang, Yong Zhi Cheng, Xian Wang, Yi Nan Zhang, Yan Nie, Rong Zhou Gong

**Affiliations:** 1 School of Optical and Electronic Information, Huazhong University of Science and Technology, Wuhan 430074, China; wfolive@sina.com (F.W.); 15927678420@163.com (Y.N.Z.); nieyan@hust.edu.cn (Y.N.); rzhgong@hust.edu.cn (R.Z.G.); 2 Engineering Research Center for Metallurgical Automation and Detecting Technology, Ministry of Education, Wuhan University of Science and Technology, Wuhan 430081, China

**Keywords:** narrow band filter, one-dimensional photonic crystals, mirror symmetric heterostructure, telecommunication wavelength

## Abstract

In this paper, we present a high-efficiency narrow band filter (NBF) based on quasi-one-dimensional photonic crystal (PC) with a mirror symmetric heterostructure. Similarly to the Fabry-Perot-like resonance cavity, the alternately-arranged dielectric layers on both sides act as the high reflectance and the junction layers used as the defect mode of the quasi-one-dimensional PC, which can be designed as a NBF. The critical conditions for the narrow pass band with high transmittance are demonstrated and analyzed by simulation and experiment. The simulation results indicate that the transmission peak of the quasi-one-dimensional PC-based NBF is up to 95.99% at the telecommunication wavelength of 1550 nm, which agrees well with the experiment. Furthermore, the influences of the periodicity and thickness of dielectric layers on the transmission properties of the PC-based NBF also have been studied numerically. Due to its favorable properties of PC-based NBF, it is can be found to have many potential applications, such as detection, sensing, and communication.

## 1. Introduction

Since photonic crystals (PCs) were initially proposed by Yablonovitch and John et al. [1,2], they have attracted significant attention due to its novel electromagnetic (EM) properties and potential applications in optoelectronic-related areas [3,4,5]. Similarly to electronic band gaps of semiconductors, PCs possess the photonic band gap (PBG) for the reason of Bragg scattering in a periodical dielectric structure [6,7]. The propagation of EM waves in PCs is strongly inhibited when the frequency falls into the PBG [8,9]. When the defect layer is introduced into PCs, the localized defect mode would appear inside the PBG due to its breaking of the periodicity [10,11,12,13]. For the defective PC, the defect mode would induce a transmission peak in the PBG region, which satisfies well the requirement of the narrow band filter (NBF). This allows the wave propagate at a frequency point while forbidding waves at all other frequency ranges, so the NBF is one of the most prevalent applications [14,15].

In practice, the conventional NBFs originate from the multi-beam interference in the Fabry-Perot-like filters [16,17], such as metal-dielectric-metal (MDM) and all-dielectric filters, which were not easy to be integrated into the current miniaturization optoelectronic system. To solve the problem, the efficient and compact NBFs were proposed based on PCs composed of multilayer films [18,19]. Some studies have been devoted to the selection of special materials of the defect layer and design novel structures for the defective PC, which could achieve the tunable and multiple high-quality NBF [20,21]. However, these designs are not convenient for application because of the complicated materials and structures, and most of them are only the simulation results. Thus, the NBF with a narrow pass band and high transmission, using accessible materials and concise structures, is highly desirable. Generally, the full width at half maximum (FWHM) and the quality factor (Q) are two main parameters for measuring the performance of a filter [22,23], the smaller FWHM and the larger Q are needed for a high-quality NBF. The relative bandwidth defined by the ratio of the FWHM to the central wavelength is used to certify a filter as broadband or narrowband [24,25]. For a NBF, the relative bandwidth is usually below 0.05 (FWHM/*λ*_0_ < 0.05) [26,27]. Further evaluation of whether a filter is an ultra-narrow bandwidth filter (UNBF) depends on its half-power bandwidth corresponding to the power with 3 dB attenuation [28,29]. When the half-power bandwidth of the filter is less than 0.001 nm (∆*λ*_3dB_ < 0.001 nm) [30,31], the filter can be regarded as an UNBF. The Q factor of the defect resonance is defined as the center wavelength divided by the 3 dB power bandwidth [32,33].

In this paper, we designed and fabricated a NBF at 1550 nm based on a defective quasi-one-dimensional PC composed of two parts of mirror-symmetric structures, each of them are constituted by the alternating arranged dielectric materials with high and low refractive indices. The proposed NBF is easily realized due to the accessible materials and concise structures, which not only provides high-reflectance in a wide wavelength range, but also achieves novel filter properties with a relatively narrow pass band. Firstly, we gave a theoretic analysis through the transfer matrix method (TMM) for a quasi-one-dimensional PC-based filter. Secondly, the simulation and experiment were performed for the designed NBF with the appropriate materials parameters and period number ((Nb_2_O_5_/SiO_2_)^6^(SiO_2_/Nb_2_O_5_)^6^). Through the mirror symmetric heterostructure design, the NBF has a high transmittance peak of 95.99% with the FWHM of 3.2 nm and Q = 705 at 1550 nm. Finally, the transmission properties of the proposed NBF were analyzed for further improvement and tuning by changing the periodicity and thickness of the multilayer films of the quasi-one-dimensional PC.

## 2. Physical Mechanism and Theoretical Derivation

**Figure 1:**
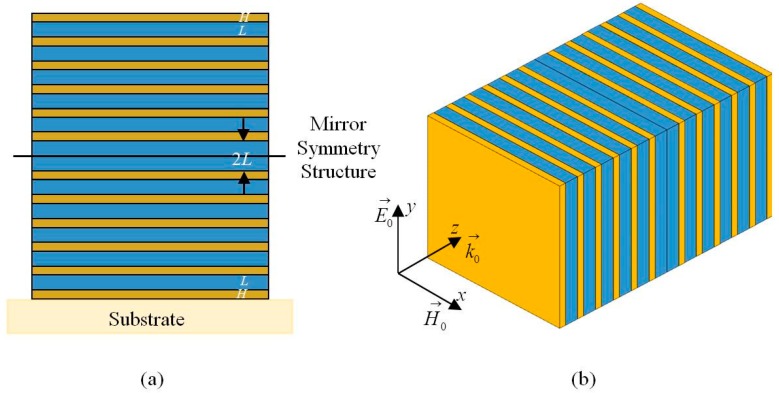
Schematic of a quasi-one-dimensional PC with the mirror-symmetric heterostructure: (**a**) lattice view; and (**b**) perspective view. Orange and blue regions denote high and low refractive index dielectric layers, respectively. The 2*L* junction region indicates the defect layer.

Figure 1 presents the schematic design of the proposed quasi-one-dimensional PC-based NBF, which consists of two identical symmetric structures compounded of two alternating dielectric layers with high and low refractive indices *n_H_* and *n_L_*, respectively. First, it is necessary to design a periodic structure, in which the propagation of EM waves is inhibited in a broad wavelength range. In this design, since the maximum range of the forbidden band can be achieved in usual quarter-wave stacks, so the optical thickness of two materials was *n_H_d_H_* = *n_L_d_L_* = *λ*_0_/4, where *d_H_* and *d_L_* are the thicknesses of high and low refraction materials, and the *λ*_0_ is central wavelength. Then, to obtain a high transmission peak located inside the center of a broad forbidden band wavelength range, the defective mode into the PC is introduced by the junction of two identical mirror symmetric structures together, which are denoted as (*HL*)*^N^*(*LH*)*^N^*, where *N* is the periodicity, and contiguous 2*L* is the defect layer. Thus, the designed quasi-one-dimensional PC-based NBF has 4*N* layers and 4*N* + 1 interfaces.

As shown in Figure 1, we assume that an EM wave with the wave vector ${\vec{k}}_{0}$ along the *z*-direction, electric field ${\vec{E}}_{0}$ along the *y*-direction (*y*-axis), and the magnetic field ${\vec{H}}_{0}$ along the *x*-direction (*x*-axis) is normally incident to the surface of the proposed quasi-one-dimensional PC. We then assume that each layer is homogeneous in the *x-y* plane and periodic in the *z*-direction, and imagine that all directions extend to infinity. Components of the electric and magnetic fields can be related through the transfer matrix as follows:

(1) $$\left(\begin{array}{c} {\vec{k}}_{0}\times {\vec{E}}_{0}\\ {\vec{H}}_{0} \end{array}\right)={\left(M_{H}M_{L}\right)}^{N}{\left(M_{L}M_{H}\right)}^{N}\left(\begin{array}{c} {\vec{k}}_{0}\times {\vec{E}}_{4N+1}\\ {\vec{H}}_{4N+1} \end{array}\right)=\left(\begin{array}{cc} A & B\\ C & D \end{array}\right)\left(\begin{array}{c} {\vec{k}}_{0}\times {\vec{E}}_{4N+1}\\ {\vec{H}}_{4N+1} \end{array}\right)$$

where:

$$M_{H}=\left(\begin{array}{cc} \cos\delta_{H} & \frac{i}{\eta_{H}}\sin\delta_{H}\\ i\eta_{H}\sin\delta_{H} & \cos\delta_{H} \end{array}\right)=\left(\begin{array}{cc} \cos\frac{\pi }{2}\cdot \frac{\lambda_{0}}{\lambda } & \frac{i}{n_{H}}\sin\frac{\pi }{2}\cdot \frac{\lambda_{0}}{\lambda }\\ in_{H}\sin\frac{\pi }{2}\cdot \frac{\lambda_{0}}{\lambda } & \cos\frac{\pi }{2}\cdot \frac{\lambda_{0}}{\lambda } \end{array}\right)$$

and:

$$M_{L}=\left(\begin{array}{cc} \cos\delta_{L} & \frac{i}{\eta_{L}}\sin\delta_{L}\\ i\eta_{L}\sin\delta_{L} & \cos\delta_{L} \end{array}\right)=\left(\begin{array}{cc} \cos\frac{\pi }{2}\cdot \frac{\lambda_{0}}{\lambda } & \frac{i}{n_{L}}\sin\frac{\pi }{2}\cdot \frac{\lambda_{0}}{\lambda }\\ in_{L}\sin\frac{\pi }{2}\cdot \frac{\lambda_{0}}{\lambda } & \cos\frac{\pi }{2}\cdot \frac{\lambda_{0}}{\lambda } \end{array}\right)$$

(*δ_H_* and *δ_L_* are the effective phase thickness, *η_H_* and *η_L_* are the effective optical admittance) are the characteristic transfer matrices determined by the properties of each layer and incident EM waves. According to the calculation method of the transmission and reflection coefficients, we can further obtain the transmittance and reflectance of the photonic band gap as follows:

(2) $$T=\frac{4n_{0}n_{4N+1}}{{\left|n_{0}\left(A+Bn_{4N+1}\right)+C+Dn_{4N+1}\right|}^{2}}$$

(3) $$R={\left|\frac{n_{0}\left(A+Bn_{2N+1}\right)-\left(C+Dn_{2N+1}\right)}{n_{0}\left(A+Bn_{2N+1}\right)+\left(C+Dn_{2N+1}\right)}\right|}^{2}$$

where *R* corresponds to the single periodic structure (*HL*)*^N^*, *T* corresponds to two identical mirror symmetric structures (*HL*)*^N^*(*LH*)*^N^*. The transfer matrix for the normally incident wave with the central wavelength (*λ* = *λ*_0_) can be calculated as:

$$\begin{array}{l} \left(\begin{array}{cc} A & B\\ C & D \end{array}\right)={\left(M_{H}M_{L}\right)}^{N}{\left(M_{L}M_{H}\right)}^{N}=\left(\begin{array}{cc} {\left(-n_{L}/n_{H}\right)}^{N} & 0\\ 0 & {\left(-n_{H}/n_{L}\right)}^{N} \end{array}\right)\left(\begin{array}{cc} {\left(-n_{H}/n_{L}\right)}^{N} & 0\\ 0 & {\left(-n_{L}/n_{H}\right)}^{N} \end{array}\right)=\left(\begin{array}{cc} 1 & 0\\ 0 & 1 \end{array}\right) \end{array}$$

and:

$$\left(\begin{array}{cc} A & B\\ C & D \end{array}\right)={\left(M_{H}M_{L}\right)}^{N}=\left(\begin{array}{cc} {\left(-n_{L}/n_{H}\right)}^{N} & 0\\ 0 & {\left(-n_{H}/n_{L}\right)}^{N} \end{array}\right),$$

respectively. Thus, Equations (2) and (3) can be simplified as follows:

(4) $$T=\frac{4n_{0}n_{4N+1}}{{\left(n_{0}+n_{4N+1}\right)}^{2}}$$

(5) $$R={\left|\frac{n_{0}{\left(-n_{L}/n_{H}\right)}^{N}-n_{2N+1}{\left(-n_{H}/n_{L}\right)}^{N}}{n_{0}{\left(-n_{L}/n_{H}\right)}^{N}+n_{2N+1}{\left(-n_{H}/n_{L}\right)}^{N}}\right|}^{2}$$

where the high transmission is determined only by the properties of the incident and exit medium, while the high reflection is dependent on not only on the outside medium, but also the alternating layers’ characteristics.

According to Equation (4), if assuming the medium of the incident wave region is air (*n*_0_ = 1), and the substrate is glass (*n*_4*N*+1_ = 1.5), the transmittance will be a constant. If the refractive index of the substrate is approximately close to the air index, then transmittance (*T*) is near unity. Furthermore, once the periodicity and refractive indices of the quasi-one-dimensional PC is defined, the reflectance will also be close to a constant. The reflectance (*R*) also can be near unity when increasing the periodicity according to Equation (5).

## 3. Simulation and Experiment

According to the above theoretical analysis, we can perform a numerical simulation using the thin-film simulation Essential Macleod (version 9.7.0, infotek, Shanghai, China) based on TMM for the proposed quasi-one-dimensional PC with a mirror symmetric heterostructure. The materials selection for the PC is very important, which is easy to be fabricated when constructing the NBF based on quasi-one-dimensional PC for application in the infrared communication region. Here the materials composed multilayer films are Nb_2_O_5_ and SiO_2_ (see Table S1 in the Supplementary Material), corresponding to the high (*H*) and low (*L*) refractive index material, respectively. To study the transmission properties of the proposed quasi-one-dimensional PC in the forbidden band, we supposed that the mirror symmetric heterostructure is (Nb_2_O_5_/SiO_2_)^6^(SiO_2_/Nb_2_O_5_)^6^. The Nb_2_O_5_ and SiO_2_ were used to fabricate the multilayer filter due to its favorable optical properties: they are high and low refractive index materials with refractive indices of 2.23 and 1.44, respectively. In the simulation, the central wavelength was *λ*_0_ = 1550 nm, the thicknesses of each layer was determined by the theory of quarter-wave stacks, so each layer thickness of Nb_2_O_5_ and SiO_2_ was 174.00 nm and 268.35 nm, respectively.

To further confirm its efficiency of the proposed quasi-one-dimensional PC-based NBF, we carried out an experimental study. Firstly, we fabricated multilayer films by using physical vapor deposition (PVD) to turn solid materials into gas and deposit the material on the substrate. Here, the thermal evaporation source is an electron beam gun assisted with ion-beam deposition; and the thickness is monitored by an optical monitor for controlling the thickness precisely. In the fabrication process, we controlled the vacuum degree, the temperature in the vacuum chamber, the workpiece disk speed, oxygen filling, the deposition rate, etc. The vacuum degree and temperature mainly maintain the purity of the thin-film material and the kinetic energy of gas atoms or molecules to reach the substrate were set as 1 × 10^−3^ Pa and 240 °C, respectively. The speed of the workpiece disk was set as 220 r/min, which mainly influences the homogeneity of the film. Commonly, oxygen filling was used to make the oxide film, and oxygen loss should be avoided to prevent compositional variation. The quantity of oxygen filling was 35 cc and 5 cc for making Nb_2_O_5_ and SiO_2_, respectively. According to the deposition mechanisms of different oxide materials, the deposition rates were 0.2 nm/s for Nb_2_O_5_ and 1.2 nm/s for SiO_2_. All of these parameters will influence the quality of the film, whose optimal values need to be selected, according to practice. The monolayer thicknesses of Nb_2_O_5_ and SiO_2_ were 174.00 nm and 268.35 nm, the thickness of the junction layer was 536.70 nm, and the total thickness of 24 layers was 5308.20 nm. Since the energy emission by an electron beam gun is limited, we employed ion-beam-assisted deposition to provide enough deposition energy. Thus, the enhanced packing density was achieved in making high-performance thin films.

## 4. Results and Discussion

**Figure 2:**
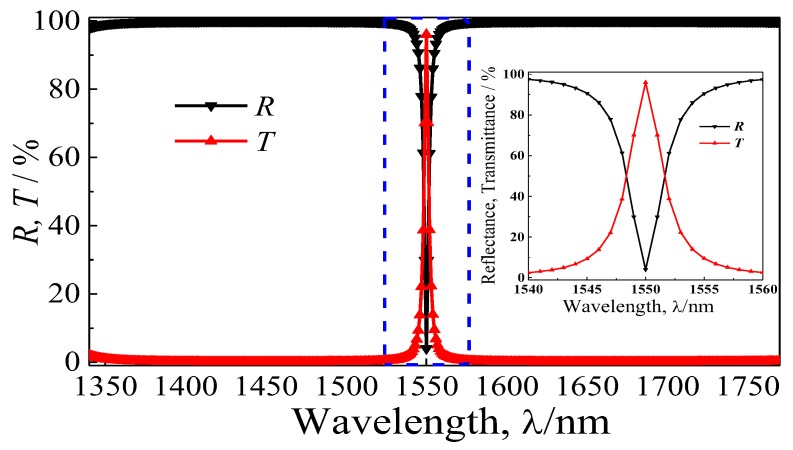
The full drawing and the inset detail enlarged drawing of the simulated reflectance (*R*) and transmittance (*T*) spectra of the designed quasi-one-dimensional PC with (Nb_2_O_5_/SiO_2_)^6^(SiO_2_/Nb_2_O_5_)^6^.

Figure 2 presents the simulation reflectance and transmittance spectra of the proposed quasi-one-dimensional PC. It can be observed that the reflection dip (*R*_min_) is decreased to about 4.01%, and the corresponding transmission peak (*T*_max_) is about 95.99% at 1550 nm (*R* + *T* = 1, the absorption is negligible in the transparent area). In addition, the FWHM (*T* = 1/2*T*_max_, ∆*λ*_h_) of the designed quasi-one-dimensional PC is 3.2 nm, thus, the high-quality Q factor (*λ*_0_/∆*λ*_3dB_) is about 705. The relative bandwidth is 0.002 (∆*λ*_h_/*λ*_0_), which is far less than the requirement of the NBF, while the half-power bandwidth of 2.2 nm (*T* = $\sqrt{2}/2$*T*_max_, ∆*λ*_3dB_) cannot meet the requirement of the UNBF, which is limited by the finite periodicity number. If the periodicity number (*N*) is up to 15, the half-power bandwidth will be below 0.001 nm; this will be confirmed in the following discussion of the periodicity. The transmission peak of the NBF fabricated with these films is limited by a mismatch of the refractive indices of the air (*n*_0_ = 1) and substrate (*n*_s_) medium. When *n*_s_ approaches approximately to 1, the *T*_max_ is near 100%. The refractive indices of the usual substrate materials, like germanium and silicon, are about 4.4 and 3.4, respectively, which are much larger than the air refractive index. While the refractive index of the common glass substrate mainly composed of SiO_2_ is about 1.5, which is much closer to 1. Thus, the *T*_max_ value is limited to 96% at the central wavelength according to Equation (4), which further confirms the simulation result. It should be noted that the transmission properties of the NBF will be changed significantly under oblique incidence (see Figure S1 and Table S2 in the Supplementary Material).

**Figure 3:**
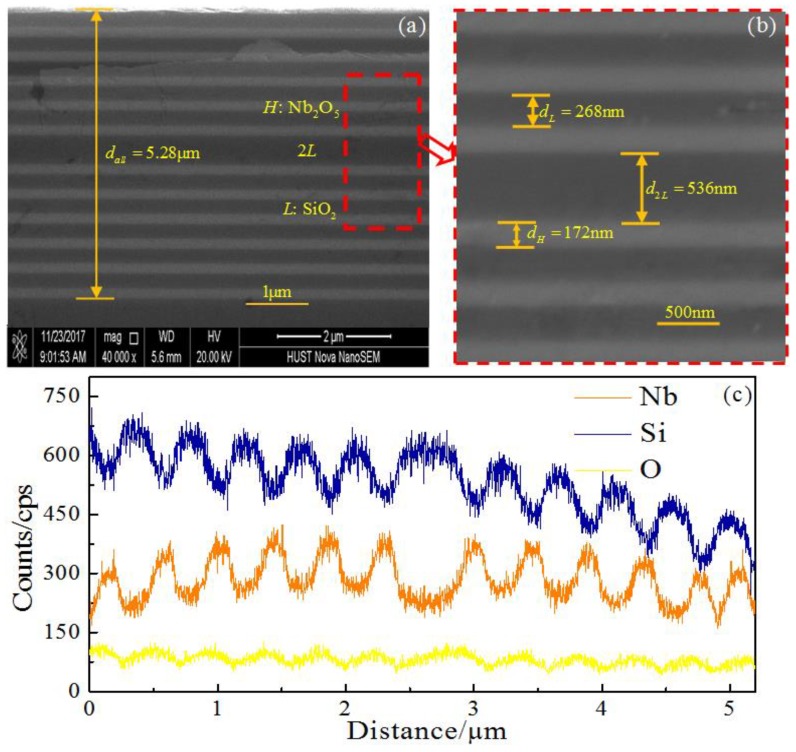
SEM image of the fabricated quasi-one-dimensional PC-based NBF cross-section: (**a**) total lattice view; (**b**) middle layers views; and (**c**) component analysis EDS diagram of each layer.

We performed the experiments according to the optimized design and numerical simulation of the proposed quasi-one-dimensional PC. A scanning electron microscope (SEM, FEI, Nova Nano SEM 450, USA) and an X-ray energy dispersive spectrometer (EDS, Oxford Instruments IncaX-Max20, United Kingdom) were used to examine microscopic cross-sections, and analyze the composition of the multilayer film, respectively. Figure 3a,b present the SEM image of cross-section of the fabricated quasi-one-dimensional PC-based NBF, where the alternating light and dark gray layers indicate the high (*H*) and low (*L*) refractive index materials of Nb_2_O_5_ and SiO_2_. The total thickness of the fabricated quasi-one-dimensional PC-based NBF is about 5.28 μm, as shown in Figure 3a. From Figure 3b, the thickness of single layers of *H* and *L* materials is 172 nm and 268 nm, respectively, and the one of 2*L* defect layer is 536 nm. It should be notied that the actual thickness of the quasi-one-dimensional PC slightly deviates from the theoretical thickness. As shown in Figure 3c, the EDS line scan is along the cross-section to identify the components of the film. The alternating content peaks indicate the main components of each layer, the orange curve represents Nb_2_O_5_, and the blue curve represents SiO_2_. The content peaks appear alternately, indicating the two kinds of materials’ alternating deposition. For the mirror-symmetric heterostructure, the junction layer as the defect layer consists of SiO_2_ (2*L*), corresponding to the broadest blue peak content in the middle of the component analysis diagram. From the middle to both sides, six orange peaks represent Nb_2_O_5_ (*H*), and five blue peaks represent SiO_2_ (*L*), respectively. It is shown that the composition of each layer coincides well with the designed structure (*HL*)^6^(*LH*)^6^.

**Figure 4:**
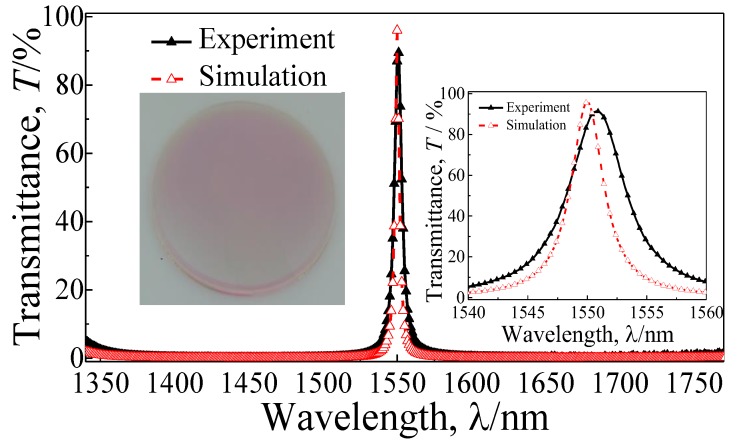
Comparative analysis of the measured and simulated transmittance (*T*). The insets show the fabricated quasi-one-dimensional PC-based NBF picture and the detailed enlarged drawing of the spectra.

Figure 4 presents the fabricated quasi-one-dimensional PC-based NBF and comparison results of the simulation and experiment. The transmission spectrum of the film is measured by the UV–VIS–NIR spectrophotometer (Shimadzu UV-3600 Plus, Shimadzu, Japan). The measured transmittance is depicted in Figure 4, which agrees reasonably well with the simulation result. However, the transmission peak is shifted slightly to the long wave direction due to the tolerances in the fabrication. There are minor discrepancies in the thickness of each layer between the simulation and measurement. The following performance parameters of the high-quality NBF by experiment can be obtained: a transmission peak of 91.37% at 1550.8 nm, the FWHM of 5.6 nm, and the Q factor of 419. Due to the narrow FWHM, the high Q factor, and the high-efficiency transmission property, it is expected that the designed quasi-one-dimensional PC-based NBF can be widely applicable in detection, sensing, and communication fields.

To better understand the physical mechanism of the observed high-efficiency transmission property of the proposed quasi-one-dimensional PC, we studied the electric field distributions at different wavelengths. Here, the incident infrared plane waves are normal to the surfaces of the film. This is a dynamic transmission process; we just select three significant wavelength points (1440 nm, 1550 nm and 1690 nm) to describe the electric field (TE) distributions. The distributions of the magnetic field (TM) are similar to the electric field (TE), due to the high symmetry of quasi-one-dimensional PC structure.

**Figure 5:**
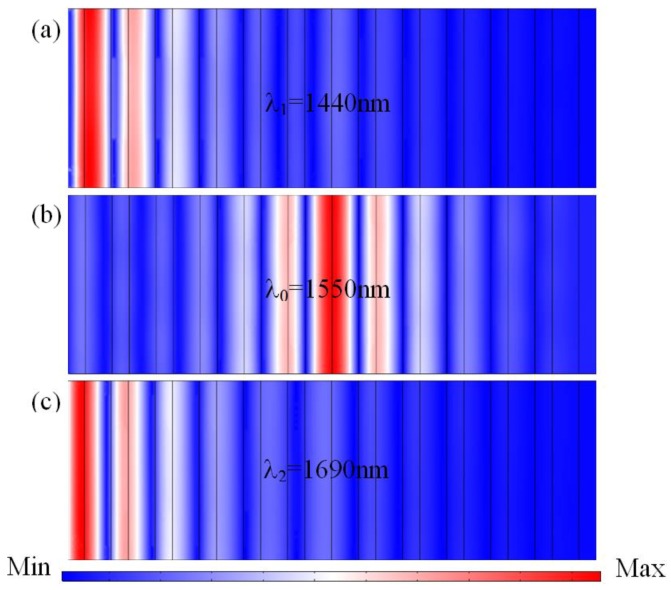
Simulated electric field distributions of the proposed quasi-one-dimensional PC-based NBF with (Nb_2_O_5_/SiO_2_)^6^(SiO_2_/Nb_2_O_5_)^6^ at different wavelengths: (**a**) *λ*_1_ = 1440 nm; (**b**) *λ*_0_ = 1550 nm; and (**c**) *λ*_2_ = 1690 nm.

According to the above simulation and experiment, the middle junction layers inducing the defect mode allow the wave of the central wavelength to propagate through the quasi-one-dimensional PC structure, and the propagation is inhibited when their wavelengths fall into the forbidden band. In Figure 5, we present the electric field distributions for the selected three wavelengths of 1440 nm, 1550 nm, and 1690 nm, respectively. As shown in Figure 5b, for the central wavelength of *λ*_0_ = 1550 nm, the electric field intensity distributions mainly focus on the middle junction layers. From Figure 5a,c, for the other two wavelengths of *λ*_1_ = 1440 nm and *λ*_2_ = 1690 nm, the electric field intensity distributions fall into the two sides of the forbidden band, and the propagation of EM waves gradually dampens in multilayer films.

**Figure 6:**
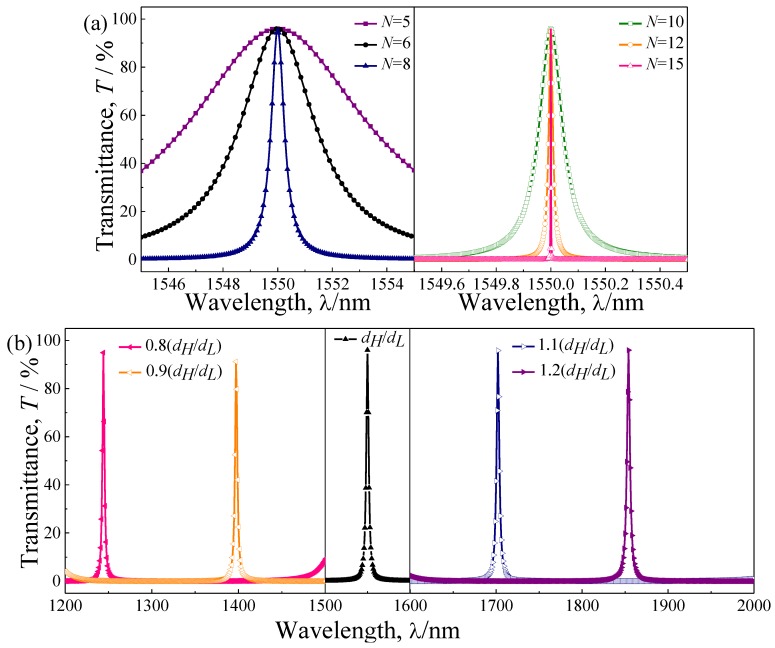
The transmission spectrum of the quasi-one-dimensional PC-based NBF with different parameters: (**a**) periodic number (*N*); and (**b**) thickness of two dielectric material layers.

We further studied, numerically, the influence of the periodicity and dielectric material thickness on the transmission spectrum of the proposed quasi-one-dimensional PC-based NBF. Firstly, the transmission spectra of the designed NBF with different periodicity *N* ((Nb_2_O_5_/SiO_2_)*^N^*(SiO_2_/Nb_2_O_5_)*^N^*, *N* = 5, 6, 8, 10, 12 and 15) were calculated when the thickness of the two dielectric material layers was at the initial thickness (*d_H_*/*d_L_* = 174.00 nm/268.35 nm). As shown in Figure 6a, it can be seen that the FWHM of the NBF decreases gradually with the increase of the periodicity, while the wavelength range and magnitude of the transmission peak is nearly unchanged with the change of the periodicity. With the increase of the periodicity, the relative bandwidth is decreased gradually and much less than 0.05, and the half-power bandwidth may be less than 0.001 nm. Although the narrowband characteristic of the NBF can be improved with the increase of the periodicity number, the conditions of fabrication should also been taken into account.

Then, we calculated the transmission spectra of the designed NBF with a different thickness of the two dielectric material layers. Setting *t_h_* as the equal scaling factor, the selected value of which is 0.8, 0.9, 1.0, 1.1, and 1.2, and the periodicity is fixed as *N* = 6. The *t_h_* = 1.0 is corresponding to the initial thickness (*d_H_*/*d_L_* = 174.00 nm/268.35 nm), the other thicknesses are scaled in respective proportions of *t_h_* = 0.8, 0.9, 1.1, and 1.2, respectively. It can be clearly observed in Figure 6b, the wavelength of transmission peak will have a significant blue-shift with increase of the thickness of two dielectric layers. However, the FWHM and Q are nearly unchanged with the change in the thicknesses of each layer. Therefore, the transmission properties of the proposed quasi-one-dimensional PC-based NBF can be tuned easily.

## 5. Conclusions

In summary, we proposed a NBF based on a quasi-one-dimensional PC with a mirror symmetric heterostructure at a near-infrared telecommunication wavelength. By introducing a mirror symmetric heterostructure, a localized defect mode appears inside the forbidden band, resulting in a high transmission at the central wavelength of the proposed quasi-one-dimensional PC. Numerical simulations indicate the transmittance peak can be up to 95.99% with a FWHM of 3.2 nm and Q factor of 705 at 1550 nm of the telecommunication wavelength, which agree reasonably well with the experiment. The quality of the NBF can be improved by changing the periodicity, and the wavelength range of transmission peak can be independently tuned by changing the thickness of two dielectric material layers. Such transmission properties of the quasi-one-dimensional PC provide an effective way for designing ultra-NBF and tunable NBF. Due to its favorable filter property of the proposed quasi-one-dimensional PC-based NBF, it can be expected that they have many potential applications in detection, sensing, and communication.

## Supplementary Materials

The following are available online at http://www.mdpi.com/1996-1944/11/7/1099/s1, Figure S1: The simulated reflectance and transmittance spectra of the designed 1DPCs with (Nb2O5/SiO2)^6^(SiO2/Nb2O5)^6^ when the incident angle is set to 10°, 20°, 30°, 40°, 50°, 60°, 70°, and 80°, Table S1: Common use materials and evaporation technical data, Table S2: The influence of different incident angles to the properties of NBF.
