# Supplementary material for: Narrow Band Filter at 1550 nm Based on Quasi-One-Dimensional Photonic Crystal with a Mirror-Symmetric Heterostructure
Source: Materials (Basel). 2018 Jun 27;11(7):1099. doi: 10.3390/ma11071099 (PMC6073759; doi:10.3390/ma11071099)
Supplement: Supplementary file 1 [file materials-11-01099-s001.pdf]

Supplementary Materials: Narrow Band Filter at 1550 nm Based on Quasi-One-Dimensional Photonic Crystal with a Mirror-Symmetric Heterostructure

Fang Wang [1], Yong Zhi Cheng [2,*], Xian Wang [1,*], Yi Nan Zhang [1], Yan Nie [1] and Rong Zhou Gong [1]

[1] School of Optical and Electronic Information, Huazhong University of Science and Technology, Wuhan 430074, China; wfolive@sina.com (F.W.); 15927678420@163.com (Y.N.Z.); nieyan@hust.edu.cn (Y.N.); rzhgong@hust.edu.cn (R.Z.G.)
[2] Engineering Research Center for Metallurgical Automation and Detecting Technology, Ministry of Education, Ministry of Education, Wuhan University of Science and Technology, Wuhan 430081, China
* Correspondence: chengyz@wust.edu.cn (Y.Z.C.); wangx@hust.edu.cn (X.W.)

Materials and Incident Angles

Table S1 to S2

Figure S1

References (33-35)

1. Materials and Incident Angles

1.1. Selection of Materials

In our design, the multilayer film is applied as the narrow band filter (NBF) similarly to the Fabry-Perot cavity. The designed NBF is based on a quasi-one-dimensional photonic crystal (PC) with a structure denoted as $(HL)^6(LH)^6$, for this defective PC, the defect mode is located inside the photonic band gap (PBG), and its spectral position with respect to the PBG center is defined by parameters of the multilayer films (thickness and refractive index). Here the materials composed of multilayer films are Nb_2O_5 and SiO_2, corresponding to the high (H) and low (L) refractive index materials, respectively. The materials selected are due to the following demands for the application of NBF in the infrared communication region:

1) Their transparency range should cover the application infrared communication band (0.8–1.8 μm), otherwise the film will absorb, thus reduces transparency.
2) These materials should be easily prepared by using advanced coating technology for achieving high-quality film.
3) Their matching degree of these two kinds of materials should be high, which will directly influence the cohesion and stability between different layers.

The common materials satisfied with the above demands are listed in Table S1. For the experiment, physical vapor deposition (PVD) was used to turn solid materials into gas and deposit them on the substrate. Here, the films were deposited by electron beam gun evaporation (EBD) and ion-beam assisted deposition (IAD) [34,35]. During the process of

vacuum coating, the electron gun produces a high-energy electron beam to directly impact the target materials sublimating the solid into gas molecules or atoms, then these gas molecules or atoms, obtaining enough energy from ion-beam assistance, travel through the vacuum chamber to deposit upon the substrate. As shown in Table S1, using EBD with IAD can obtain a high stacking density of Nb_2O_5, which always matches with SiO_2 to fabricate multilayer films due to its small stress. It is well known that the greater the difference between the high and low refractive indices, the wider the forbidden band and the narrower the transmission peak that can be obtained. TiO_2 and Ta_2O_5 as oxide films also have high refractive indices; however, compared with Nb_2O_5, the quality of the NBF composited with SiO_2 will decline slightly. MgF_2 and Na_3AlF_6, as fluoride films have low refractive indices. However, due to stress problems, MgF_2 always matches with ZnS to prevent film cracking, and the low firmness of Na_3AlF_6 is not resistant to the environment. Considering the quality of the filter and the preparation technology, we selected Nb_2O_5 and SiO_2 as suitable materials.

1.2. Angular Dependence for Quasi-One-Dimensional PC-Based NBF

In the simulation, the incident wave is of linear polarization, the TE wave has an electric field parallel to the interface and is named as S polarization, while the TM wave with a magnetic field parallel to the interface is named as P polarization. We considered only the condition of light normally incident to the surface of the proposed quasi-one-dimensional PC in the paper, where the S and P polarizations are identical. For further discussion of angular dependence caused by oblique incidence [36,37], we present the transmittance and reflectance of the proposed quasi-one-dimensional PC with different incident angles, θ, changed from 0° to 80°. As shown in Figure S1, it can be observed that the wavelength of the transmission peak and forbidden band is blue-shifted significantly with the increase of the incident angle. Furthermore, S-pol and P-pol (dashed and solid lines) exhibit polarization that gradually separates with the increase of the incident angle. When the angle of incidence increases from 0° to 80°, the detailed data of the properties of the quasi-one-dimensional PC-based NBF are listed in Table S2. For the light of S-Pol, the magnitude of the transmission peak gradually decreases; the central wavelength shift is in the direction of short waves, and the FWHM will become narrower and the Q factor will become larger. For the light of P-Pol, the magnitude of the transmission peak increases firstly, and then decreases, and the central wavelength shift to the direction of short waves; the FWHM will become wider and the Q factor will become smaller. The value of the transmission peak, FWHM and Q factor vary with the increase of incidence angles evidently. There is a serious effect of the oblique incident angle to the performance of the NBF. In practical application, the designed quasi-one-dimensional PC for its miniaturization and integration can be loaded directly into the device port, thus, light usually has perpendicular incidence to the surface. Thus, we discussed only the performance of NBF under normal incident light.

Table S1. Common use materials and evaporation technical data.

Material	Transparency Range(μm)	Refractive Index (λ=1550nm)	Evaporating Temperature(℃)	Stress Analysis	Firmness
Nb_2O_5	0.38-8	2.23	1700		H
TiO_2	0.4-10	2.18	1700	C	H
Ta_2O_5	0.35-10	2.1	2000		H
SiO_2	0.2-8	1.44	2000	C	H
MgF_2	0.15-8	1.38	1100	T	H
Na_3AlF_6	0.2-14	1.35	1000	T	S

Note: T: tensile stress, C: compressive stress; S: soft, H: hard.

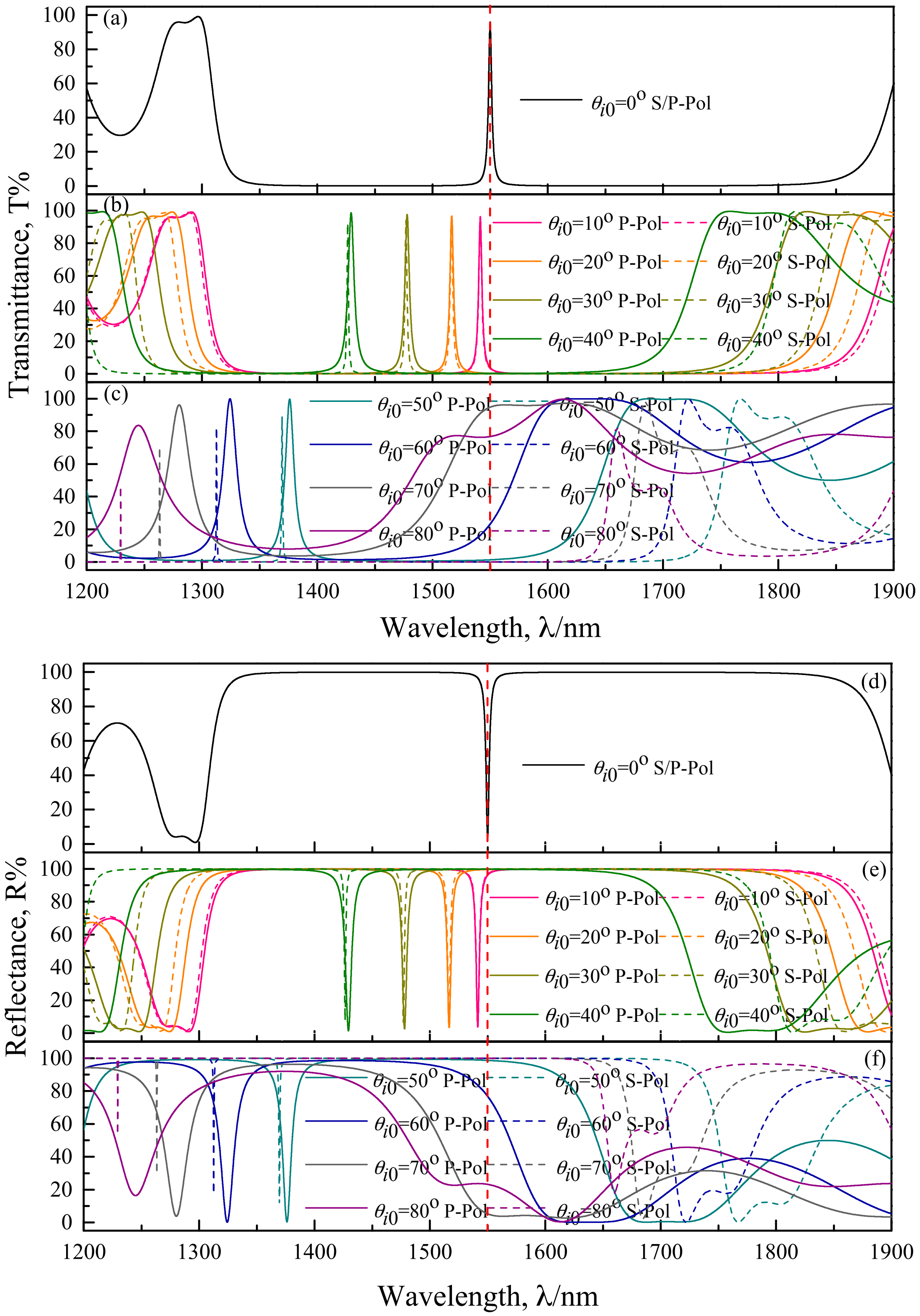

Figure S1. The simulated reflectance and transmittance spectra of the designed 1DPCs with $(Nb_2O_5/SiO_2)^6(SiO_2/Nb_2O_5)^6$ when the incident angle is set to 10°, 20°, 30°, 40°, 50°, 60°, 70°, and 80°.

Table S2. The influence of different incident angles to the properties of NBF.

θ_{i0}	Polarization	Transmission Peak T_{max} (λ nm)	FWHM (nm)	Q
0°	S/P	95.99% (1550)	3.2	705
10°	S	95.82% (1541)	3.1	771
	P	96.15% (1541)	3.4	734
20°	S	95.28% (1516)	2.7	798
	P	96.64% (1517)	3.9	607
30°	S	94.20% (1477)	2.1	1136
	P	97.47% (1478)	4.8	493
40°	S	92.27% (1426)	1.5	1585
	P	98.56% (1429)	6.4	357
50°	S	88.81% (1370)	0.9	2283
	P	99.67% (1376)	9.2	237
60°	S	82.44% (1313)	0.5	4375
	P	99.83% (1324)	14.2	144
70°	S	70.26% (1263)	0.4	6317
	P	96.17% (1280)	24	83
80°	S	46.43% (1229)	0.3	12294
	P	83.61% (1245)	46.5	42

Note: Red: rising trend; blue: declining trend.

References

1. Yablonovitch E 1987 Inhibited spontaneous emission in solid-state physics and electronics Phys. Rev. Lett. **58** 2059.

2. John S 1987 Strong localization of photons in certain disordered dielectric superlattices Phys. Rev. Lett. **58** 2486.

3. Noda S, Tomoda K, Yamamoto N, and Chutinan A 2000 Full three-dimensional photonic bandgap crystals at near-infrared wavelengths Science **289** 604.

4. Fleming J G, Lin S Y, El-Kady I, Biswas R and Ho K M 2002 All-metallic three-dimensional photonic crystals with a large infrared bandgap Nature **417** 52.

5. Bendickson J M, and Dowling J P 1996 Analytic expressions for the electromagnetic mode density in finite, one-dimensional, photonic band-gap structures Phys. Rev. E **53** 4107.

6. Xu K Y, Zheng X G, Li C L and She W L 2005 Design of omnidirectional and multiple channeled filters using one-dimensional photonic crystals containing a defect layer with a

negative index Phys. Rev. E **71** 066604.

7. Chen Y H 2009 Tunable omnidirectional multichannel filters based on dual-defective photonic crystals containing negative-index materials J. Phys. D: Appl. Phys. **42** 075106.

8. Qi D, Wang X, Cheng Y Z, Gong R Z and Li B W 2016 Design and characterization of one-dimensional photonic crystals based on ZnS/Ge for infrared-visible compatible stealth applications Opt. Mater. **62** 52.

9. Wang X, Qi D, Wang F, Cheng Y Z, Nie Y and Gong R Z 2017 Design and fabrication of energy efficient film based on one-dimensional photonic band gap structures J. Alloy. Compd. **697** 1.

10. Liang G Q, Han P and Wang H Z 2004 Narrow frequency and sharp angular defect mode in one-dimensional photonic crystals from a photonic heterostructure Opt. Lett. **29** 192.

11. Němec H, Duvillaret L and Garet F 2004 Thermally tunable filter for terahertz range based on a one-dimensional photonic crystal with a defect J. Appl. Phys. **96** 4072.

12. Rostam M oradian, and Jamileh Samadi. Frequency comparison of light transmission in a defected quasi-one-dimensional photonic crystal slab, International Nano Letters, 2013, 3:27

13. Lee K J, Wu J W and Kim K 2014 Defect modes in a one-dimensional photonic crystal with a chiral defect layer Opt. Mater. Express **4** 2542.

14. Shi X, Xue C H, Jiang H T and Chen H 2016 Topological description for gaps of one-dimensional symmetric all-dielectric photonic crystals Opt. Express **24** 18580.

15. Villa F and Gaspar-Armenta J A 2004 Photonic crystal surface modes: narrow-bandpass filters Opt. Express **12** 2338.

16. Li Y Z, Qi L M, Yu J S, Chen Z J, Yao Y and Liu X M 2017 One-dimensional multiband terahertz graphene photonic crystal filters Opt. Mater. Express **7** 1228.

17. Jiang S J, Li J R, Tang J J and Wang H Z 2006 Multi-channel and sharp angular spatial filters based on one-dimensional photonic crystals Chin. Opt. Lett. **4** 605.

18. Zhang Y P, Gao Y and Zhang H Y 2012 Independent modulation of defect modes in fractal potential patterned graphene superlattices with multiple defect layers J. Phys. D: Appl. Phys. **45** 055101.

19. Kong X K, Liu S B, Zhang H F and Li C Z 2010 A novel tunable filter featuring defect mode of the TE wave from one-dimensional photonic crystals doped by magnetized plasma Phys.

Plasmas **17** 103506.

20. Jiang H T, Chen H, Li H Q, Zhang Y W and Zhu S Y 2005 Compact high-Q filters based on one-dimensional photonic crystals containing single-negative materials J. Appl. Phys. **98** 013101.

21. Wang H T, Lin J D, Lee C R and Lee W 2014 Ultralow-threshold single-mode lasing based on a one-dimensional asymmetric photonic bandgap structure with liquid crystal as a defect layer Opt. Lett. **39** 3516.

22. Dolgova T V, Maidykovski A I, Martemyanov M G, Fedyanin A A and Aktsipetrov O A 2002 Giant optical second-harmonic generation in single and coupled microcavities formed from one-dimensional photonic crystals J. Opt. Soc. Am. B **19** 2129.

23. Bayindir M, Kural C and Ozbay E 2001 Coupled optical microcavities in one-dimensional photonic bandgap structures J. Opt. A: Pure Appl. Opt. **3** S184.

24. Qi L, Yang Z and Fu T 2012 Defect modes in one-dimensional magnetized plasma photonic crystals with a dielectric defect layer Phys. Plasmas **19** 012509.

25. Hung H C, Wu C J and Chang S J 2011 Terahertz temperature-dependent defect mode in a semiconductor-dielectric photonic crystal J. Appl. Phys. **110** 093110.

26. Schmidt R V, Flanders D C, Shank C V and Standley R D 1974 Narrow-band grating filters for thin-film optical waveguides Appl. Phys. Lett. **25** 651.

27. Alferness R C, Joyner C H, Divino M D, Martyak M J R and Buhl L L 1986 Narrowband grating resonator filters in InGaAsP/InP waveguides Appl. Phys. Lett. **49** 125.

28. Chen G B and Yu H C 2014 The enlargement of high reflectance range in ultra-narrow bandpass filter with disordered one-dimensional photonic crystal J. Appl. Phys. **115** 033114.

29. Li Y, Xiang Y J, Wen S C, Yong J H and Fan D Y 2011 Tunable terahertz-mirror and multi-channel terahertz-filter based on one-dimensional photonic crystals containing semiconductors J. Appl. Phys. **110** 073111.

30. Wu C J and Wang Z H 2010 Properties of defect modes in one-dimensional photonic crystals Prog. Electromagn. Res. **103** 169.

31. Lotfi E, Jamshidi-Ghaleh K, Moslem F and Masalehdan H 2010 Comparison of photonic crystal narrow filters with metamaterials and dielectric defects Eur. Phys. J. D **60** 369.

32. Mao D, Ouyang Z, Wang J C, Liu C P and Wu C J 2008 A photonic-crystal polarizer

integrated with the functions of narrow bandpass and narrow transmission-angle filtering Appl. Phys. B-Lasers O. **90** 127.

33. Hawkeye M M and Brett M J 2006 Narrow bandpass optical filters fabricated with one-dimensionally periodic inhomogeneous thin films J. Appl. Phys. **100** 044322.

34. Colodrero S, Ocaña M, González-Elipe A R and Míguez H 2008 Response of Nanoparticle-Based One-Dimensional Photonic Crystals to Ambient Vapor Pressure Langmuir **24** 9135.

35. Mauricio E. C, Silvia C, Nuria H and Gabriel L 2011 Porous one dimensional photonic crystals: novel multifunctional materials for environmental and energy applications Energy Environ. Sci. **4** 4800.

36. Jiang H T, Chen H, Li H Q, Zhang Y W and Zhu S Y 2003 Omnidirectional gap and defect mode of one-dimensional photonic crystals containing negative-index materials Appl. Phys. Lett. **83** 5386.

37. Ha Y K, Yang Y C, Kim J E and Park H Y 2001 Tunable omnidirectional reflection bands and defect modes of a one-dimensional photonic band gap structure with liquid crystals Appl. Phys. Lett. **79** 15.
